# Preliminary study of the oral mycobiome of children with and without dental caries

**DOI:** 10.1080/20002297.2018.1536182

**Published:** 2018-10-23

**Authors:** Jacquelyn M. Fechney, Gina V. Browne, Neeta Prabhu, Laszlo Irinyi, Wieland Meyer, Toby Hughes, Michelle Bockmann, Grant Townsend, Hanieh Salehi, Christina J. Adler

**Affiliations:** a Discipline of Pediatric Dentistry, Westmead Centre for Oral Health, University of Sydney, Sydney, Australia; b Institute of Dental Research, School of Dentistry, Faculty of Medicine and Health, University of Sydney, Sydney, Australia; c Molecular Mycology Research Laboratory, Centre for Infectious Diseases and Microbiology, Westmead Hospital, Faculty of Medicine and Health, Westmead Clinical School, Marie Bashir Institute for Infectious Diseases and Biosecurity, The University of Sydney, Sydney, Australia; d Westmead Institute for Medical Research, Sydney, Australia; e Adelaide Dental School, Faculty of Health and Medical Sciences, The University of Adelaide, Adelaide, Australia

**Keywords:** Dental caries, fungi, oral microbiome, mycobiome, ITS

## Abstract

Children’s oral health is in a dire state, with dental decay (caries) being one of the most common chronic diseases. While the role of bacteria in the oral microbiome and dental caries is established, the contribution of fungi is relatively unknown. We assessed the oral mycobiome in childhood (*n* = 17), to determine if the composition of fungi varies between children with and without caries. Oral mycobiome composition was assessed by using Illumina MiSeq to sequence the ITS2 region, which was amplified from dental plaque. This revealed that the oral mycobiome in the investigated children contained 46 fungal species. *Candida albicans *was the most abundant species and was ubiquitous in all samples, indicating this species may not be involved in caries development as previously suggested. While the overall diversity of fungi was similar, independent of caries status (*p* > 0.05), we found caries influenced the abundance of specific fungi. Children without caries had a significantly higher abundance of 17 species compared to children with caries, which had three enriched species (*p* < 0.001). While the differentially abundant species between health and caries may be specific to an Australian population, our findings indicate the mycobiome plays a role in oral health.

## Introduction

Dental caries, one of the most preventable chronic diseases worldwide, has an extremely high prevalence in children, especially within certain socially disadvantaged populations [1–3]. In Australia, 55.7% of children aged 6–8 years and 63% aged 9 years bear the burden of this chronic condition [3]. The costs and implications of dental caries on healthcare systems are huge, resulting in an enormous burden for both children and their families. Because of this, prevention and early intervention are crucial in caries management. This, however, relies on the development of a greater understanding of the microorganisms present in the oral cavity, as the findings of new studies suggest.

The role of bacteria in caries initiation and progression is well recognised and a significant amount of literature has implicated certain bacteria as the major etiologic agents [4–8]. Recent research has revealed the aetiology of caries to be more complicated than previously thought [7,9]. Genetic analysis has shown that rather than caries being caused by a single bacterium (*Streptococcus mutans*), it is a polymicrobial disease associated with an overall change in the composition of the oral microbiome [10]. The oral microbiome is defined as ‘all the microorganisms that are found on or in the human oral cavity and its contiguous extensions such as those on the tonsils and pharynx’ [11]. However, most research investigating the oral microbiome focuses on bacteria only [12–14], with over 700 bacterial taxa known to be present in the oral cavity of humans [15]. Currently, little is known about the contribution of fungi to the oral microbiome and their role in oral health as well as dental caries.

Culture and genomics-based studies have consistently found the oral mycobiome to be dominated by *Candida albicans*. This fungus has been shown to be responsible for *Candida*-induced oropharyngeal thrush in newborns, and mucositis in denture-wearers and immunocompromised patients [16–20]. In addition to *Candida*, over 100 fungal species have been found in the oral cavity of healthy adults, including *Malassezia globosa* [21,22] and *Rhodotorula mucilaginosa* [23], using Next Generation Sequencing (NGS) [24] of the primary fungal DNA barcode, the internal transcribed spacer (ITS) region [25]. Sequencing of the ITS region enables identification of a broad range of fungi, including yeasts and moulds [21]. Application of NGS targeting the ITS region has yet to be applied to understanding the role of fungi in the development of the oral microbiome and their contribution to develop caries in childhood.

Fungi are thought to play a role in caries, given the high levels of *Candida* species found in children with early childhood caries [26–29]. It is not known, however, whether this association is direct or whether it is linked to poor oral hygiene, excessive sugar consumption and/or bottle feeding practices [26]. Current literature demonstrates that fungi can participate in biofilm structure and function and subsequently may contribute to development of caries [30]. Fungi are able to adhere to an array of surfaces, produce multiple catabolites and interfere with the immunological system of the host [31]. *C. albicans* is aciduric and acidogenic, explained by the fungal H+-ATPase which allows for a high acid tolerance and an ability to excrete organic acid subsequently further reducing the pH [32]. *C. albicans* also has the ability to break down sucrose, glucose and lactose [33]. Additionally, *Candida* species are able to invade dentinal tubules and bind to denatured collagen and secrete aspartyl proteases resulting in demineralisation and dissolution of the dental hard tissues [34]. They have a greater ability to dissolve hydroxyapatite when compared to *S. mutan*s [35]. Studies in rats have suggested that *Candida* species can cause caries when the animals are inoculated with *C. albicans* and fed a high sucrose or glucose diet [36]. By describing the fungi present in the oral microbiome, there is the opportunity to gain insights into the role of fungi, in particular *Candida* species, in dental caries.

This study is the first to investigate fungi as part of the oral microbiome in children using NGS, and in a preliminary fashion the contribution of fungi to dental caries. The aim was to identify what fungi were present in the oral microbiome in children in the mixed dentition and additionally, whether this differs between children with good oral health and those with dental caries.

## Material and methods

### Population

To determine the composition of fungi present in the oral microbiome in children, dental plaque samples were taken from 17 healthy Australian children, aged between 7 and 10 years, who had a mixed dentition. Written informed consent was obtained from the parents of all participants and ethical approval for the study was obtained from the University of Adelaide HREC (H-2013–097). The study participants included seven females and 10 males. Six participants had caries and five participants had restorations present. The gender, age, oral health and restorative status of each study participant can be found in Supplementary material 1. All 17 children were found to have fungi present in their plaque samples following PCR.

### Dental plaque sampling

Dental plaque samples were taken from each study participant. All participants had been instructed to avoid tooth brushing from 7 pm the evening prior to their appointment and were also asked to refrain from eating or drinking in the half hour prior to the appointment. Each study participant had one dental plaque sample obtained from the supra-gingival aspects of the teeth in quadrant three, which were all completed by a registered dentist. A separate sterile Catch-All sample collection swab was passed over the labial/buccal tooth surfaces and gingival margins of quadrant three for 30 s. After sampling, the swab was placed in Bead Solution (MoBio), swirled in the solution continuously for 30 s and removed. The bead solution tube was then immediately placed on dry ice, and then stored at −80°C until DNA extraction.

### Caries assessment

Concurrently with plaque-sampling, the entire dentition of each participant was assessed using the International Caries Detection and Assessment System (ICDAS II) [37]. The ICDASII is used to assess and define dental caries at the initial and early enamel lesion stages through to dentinal and finally stages of the disease. It enables reliable, informative and comparative data that are widely used for population health and clinical studies. Examiners were experienced clinicians who had undergone rigorous calibration and were routinely recalibrated across measurement sites to minimise error. For this preliminary study, each participant was subsequently allocated to one of two groups – individuals who were completely caries-free on a whole-mouth basis, or individuals with any evidence of active or previous caries experience on one or more tooth surfaces.

### Genetic analysis

Genetic analysis of the dental plaque samples included DNA extraction, amplification of the ITS2 region by PCR and sequencing of the samples with Illumina MiSeq to examine the microbial contents.

#### DNA extraction

All biofilm samples were extracted using the PowerSoil^TM^ DNA Isolation Kit (Oiagen) according to the manufacturer’s instructions, with the addition of a 10-minute incubation step at room temperature before the final centrifugation and elution step to increase DNA recovery. All samples were co-extracted with blanks to monitor for contamination.

#### Amplification of the ITS region

PCR was used to amplify the ITS2 region using the fITS7 [38] and ITS4 [39] primers. The forward primer sequence (fITS7) used was GTGARTCATCGAATCTTTG, and the reverse primer sequence (ITS4), was TCCTCCGCTTATTGATATGC. The PCR conditions included 0.5 U HotMaster Taq (QuantaBio) in a 25 μl volume using 10 x HotMaster Taq Buffer, 200 μM of each dNTP (Promega), 0.2 μM of primer and 4 μL of DNA extract. The thermocycling conditions consisted of an initial enzyme activation step at 94°C for 2 min, followed by 35 cycles of denaturation at 94°C for 20 s, annealing at 55°C for 10 s and elongation at 65°C for 40 s, with a final extension at 65°C for 10 min. Each set of PCRs included extraction and PCR blanks. All PCR products were visually examined by electrophoresis on 2.0% agarose TAE gels.

#### Illumina sequencing

This was used to examine the fungal contents of the 17 dental plaque samples DNA extracts. Amplicons were sequenced on the Illumina MiSeq platform with 250 base pair, paired-end read chemistry.

### Sequence analysis

Raw sequencing reads were processed using the USEARCH 10.0 sequence analysis tool following the denoising pipeline [40]. The steps included: (1) merging paired reads (forward and reverse) into a single consensus sequence containing one sequence with one set of quality scores, (2) assigning sample name to read labels, (3) striping primers, discarding short reads and trimming sequences to 250 bp, (4) pooling all samples together into one fastq file, (5) quality filtering by discarding reads with ambiguous and low-quality base calls, (6) detecting and removing chimeras, (7) removing singletons which are likely to be sequencing errors, (8) finding unique read sequences and abundances, (9) running UNOISE algorithms to identify all correct biological sequences in the reads with zero clustering distance [41], (10) creating an OTU table, and (11) normalising the OTU table counts to the same number of reads per sample. The most abundant sequence of each OTU was selected as a representative for taxonomic assignments using the Wang approach with 1,000 bs iterations implemented in classfy.seqs command in MOTHUR and for BLASTn searches against the most recent combined (available on 04.03.2017) UNITE full dataset [42] and ISHAM-ITS database [43].

The sequence data have been deposited with the European Nucleotide Archive with accession number PRJEB28833.

### Statistical analysis

Statistical analysis of the quality filtered and classified sequence data was undertaken in R (ver.3.4.1), using the following packages; Phyloseq (ver. 1.20.0) [36], MetagenomeSeq (ver. 1.18.0) [44] and Microbiome (ver. 1.1.1) [45].

#### Alpha diversity

Within-sample diversity was estimated per sample on quality-filtered data, which had not been submitted to any further pre-processing, such as removal of singletons. The α-diversity metrics, Chao1, Simpson’s Index and Shannon, were calculated for all samples (Phyloseq). To assess the impact of oral health status on α-diversity, while controlling for age, sex and restorations, we used an ANCOVA in R with the following formula lm(α-diversity metrics ~ Age + Sex + Restorations + Clinical_Status), with a separate model for each α-diversity metric assessed.

#### Pre-processing of sequences

Before undertaking further statistical analyses, very low abundant sequences were removed in-line with current recommendations [46]. We removed OTUs that were singletons and had abundance below 0.01% of all the sequences.

#### Normalisation of OTU count data

To account for variation in sequence depth between samples, we used Cumulative Sum Scaling (CSS) to produce normalised sequence data by library size with the MetagenomeSeq R package. All abundances of OTUs reported are from the CSS data.

#### Beta-diversity

The shared sample diversity based on CSS normalised OTU data was calculated using the Brays-Curtis distance measure. Distances were displayed graphically using non-metric dimensional scaling. A permutation-based anova (PERMANOVA) was used to assess if there was a difference in shared diversity of individuals with and without caries, in which permutations were constrained by past caries status (e.g. restoration status).

#### Core mycobiome in both health and caries

We determined an OTU to be a core member of the oral mycobiome if it was present in at least 50% of samples at a relative abundance of 0.2% in-line with other studies [22,47]. We calculated the core OTUs present in both health and caries using the CSS data with the Microbiome R package.

#### Differential OTU test

The DESeq2 package [48,49] was used to test for the presence of differentially expressed Operational Taxonomic Units (OTU’s) between the caries active and caries free individuals in R using physloseq on non-transformed data. The test is a negative binomial generalised linear model (GLM), Wald statistic and was used to model the counts of OTU’s per sample using a negative binomial distribution. The experimental design for the test was set to compare oral health (Clinical_status), while taking into account variations between samples in age, sex and past caries treatment (restorations) using the following formula phyloseq_to_deseq2(Fungi_biom_map_fil_200818, ~Sex + Age + Restorations + Clinical_Status). All OTU’s that significantly (alpha = 0.01) differed in abundance between the caries active and caries-free individuals according to the DESeq test were reported. All reported *p*-values were adjusted for multiple comparisons using the Benjamini-Hochberg, False Discovery Rate procedure. This was performed in R/phyloseq on non-transformed data, using the complete dataset and only samples with over 10,000 sequences per sample.

## Results

In-depth genetic sequencing was used to analyse the oral mycobiome composition in 17 children (7–10 years old). The ITS2 region was amplified from supragingival dental plaque samples. Illumina MiSeq sequencing of the partial ITS region produced an average of 37,119 (minimum 6,761–maximum 71,058) sequences per sample. There was a large variation in the number of sequences per sample, which did not appear to be based on clinical status (see Supplementary Figure 1).

### Childhood oral mycobiome composition

**Figure 1.**
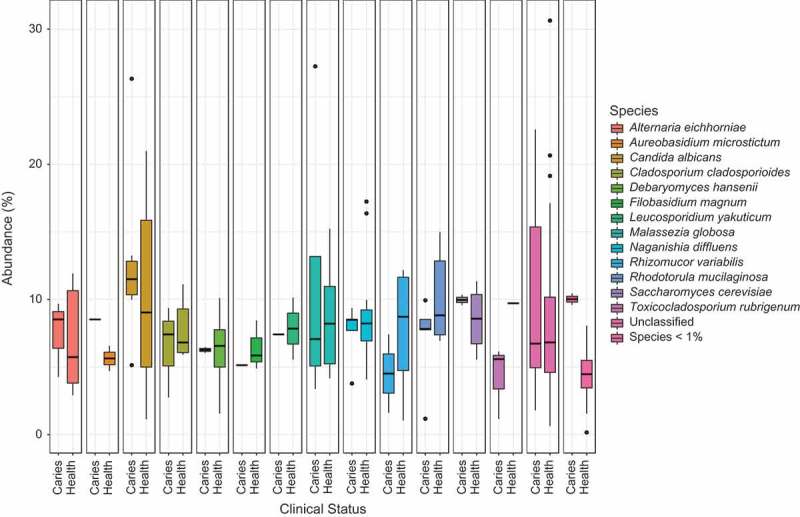
Species abundance based on clinical status. Species with a relative abundance above 1% are displayed, with the remainder of species grouped together (Species < 1%). The data have been normalised for sequence number via cumulative sum scaling (CSS).

Three fungal phyla were identified in the plaque samples (see Supplementary Figure 2 and Supplementary Material 2). These were Ascomycota, Basidiomycota and Zygomycota, with Ascomycota and Basidomycota being the most dominant phyla. In all samples, at least 84% of the fungi could be assigned a phylum, and over 50% of sequences could be identified at genus level (see Supplementary Material 2). A total of 23 fungal genera and 46 fungal species were identified across all plaque samples. Of these 46 species, 15 accounted for over 75% of the sequences (see Figure 1 and Supplementary Material 2). The most dominant species was *C. albicans* (12%, SD ± 7%), followed by *Naganishia diffluens* (8%, SD ± 4%), *R. mucilaginosa* (8%, SD ± 4%) and *M. globosa* (6%, SD ± 7%). The mean number of species per sample was 13, ranging from six to 23 species per sample (see Supplementary Material 2).

### Oral mycobiome in health and caries

**Figure 2.**
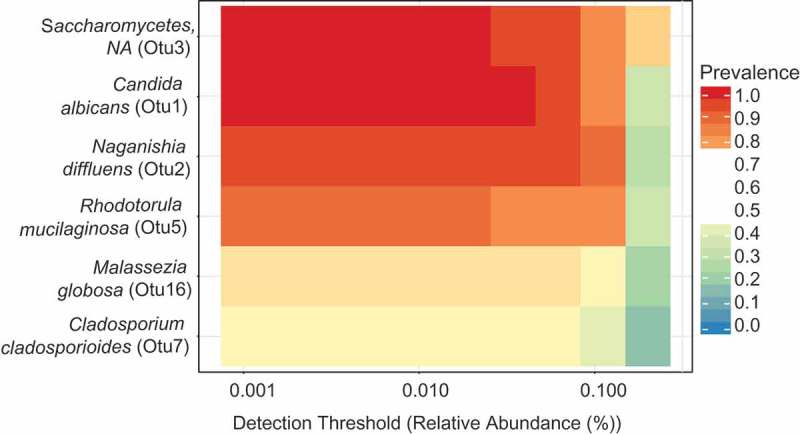
Core OTUs in health and caries. This figure shows the distribution of species present in at least 50% of samples in either health or caries at a minimum relative abundance of 0.2%.

We assessed whether there were ‘core’ fungi present in individuals with and without caries. Six OTU’s were present in at least 50% of samples with either healthy dentitions or caries (see Figure 2 and Table 1). Of these six, *C. albicans* (OTU 1) and an unclassified ***S****accharomycetes* species were present in all plaque samples. The next most ubiquitous core member in both caries and healthy dentitions was *N. _diffluens* (OTU 2), followed by *R. mucilaginosa* (OTU 5) and *M. globosa* (OTU 23). *Cladosporium cladosporoides* (OTU 7) was present in 53% of all samples.

**Table 1.** Core mycobiome OTUs.

Taxa	Prevalence (%)
p__Ascomycota__ c__Saccharomycetes__spp_Otu3	100
p__Ascomycota__s__*Candida_albicans*_Otu1	100
p__Basidiomycota__s__*Naganishia_diffluens*_Otu2	94
p__Basidiomycota__s__*Rhodotorula_mucilaginosa*_Otu5	88
p__Basidiomycota_s__*Malassezia_globosa*_Otu16	59
p__Ascomycota_s__*Cladosporium_cladosporioides_*Otu7	53

The species contributing to Figure 2 are presented in Table 1. Core OTUs were taxa found at over 50% prevalence at .2% relative abundance.

**Figure 3.**
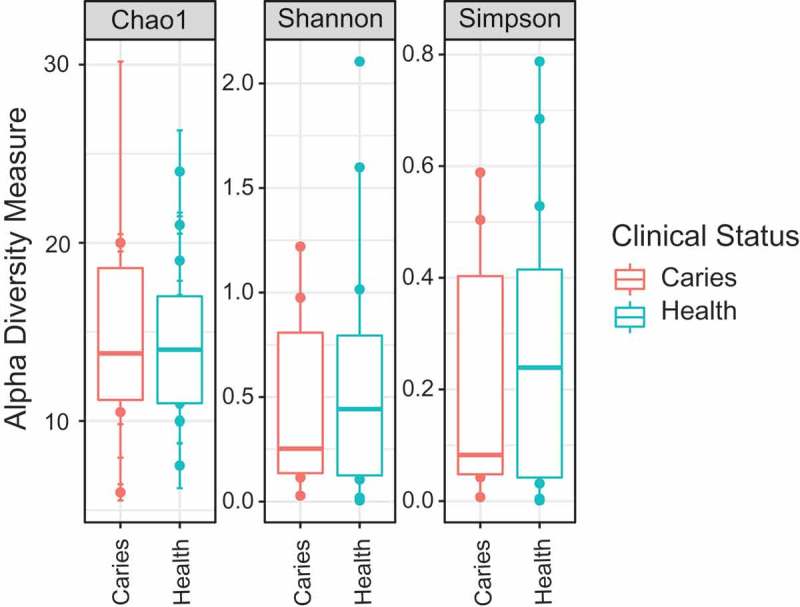
Alpha diversity (chao, simpson and shannon) by clinical status. Alpha diversity metrics were calculated in Phyloseq (version 1.20.0) and displayed as boxplots, which show the mean and 95% confidence intervals for the data.

We assessed whether the overall fungal diversity between samples differed between children with healthy dentitions and those with dental caries. Alpha (α) diversity metrics, Chao1, Simpson and Shannon, were used to measure the richness and evenness of OTUs in children in the two health states (see Figure 3). An Analysis of Covariance (ANCOVA) was conducted to determine whether there was a statistically significant effect of oral health state on the childrens’ oral mycobiome using Chao1, Simpson and Shannon values, when controlling for age, sex and the presence of past caries treatment (restorations) (Supplementary Material 3). Both analyses found no significant relationship between the richness or evenness of the overall oral fungal community and caries/healthy dentitions.

**Figure 4.**
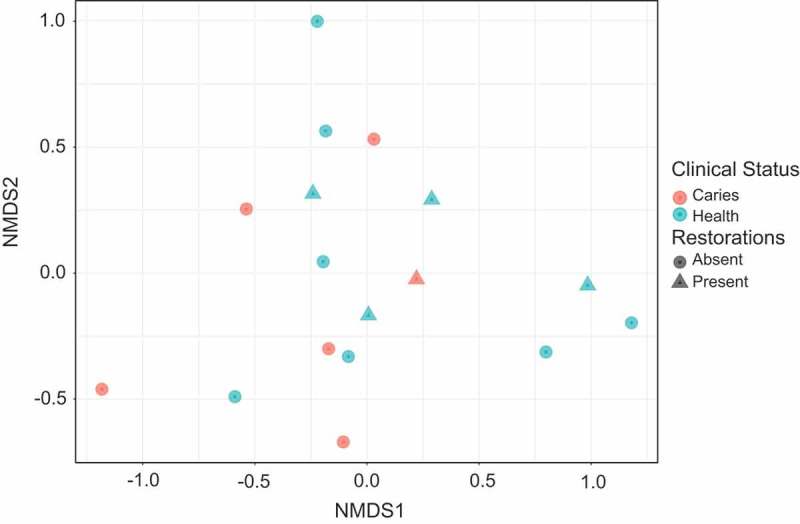
Beta diversity by clinical status. Beta diversity was calculated from CSS normalised OTU data using the Brays-Curtis distance measure and displayed using non-metric dimensional scaling (NMDS). Samples are coloured by clinical status, with past caries status indicated by shape.

Similarly, no difference was observed in the shared or beta diversity between samples based on clinical status (Figure 4). A permanova of the brays-curtis distances between individuals with and without caries, stratified by restorations, revealed no significant difference in the diversity between the two groups (*p* = 0.66)

**Figure 5.**
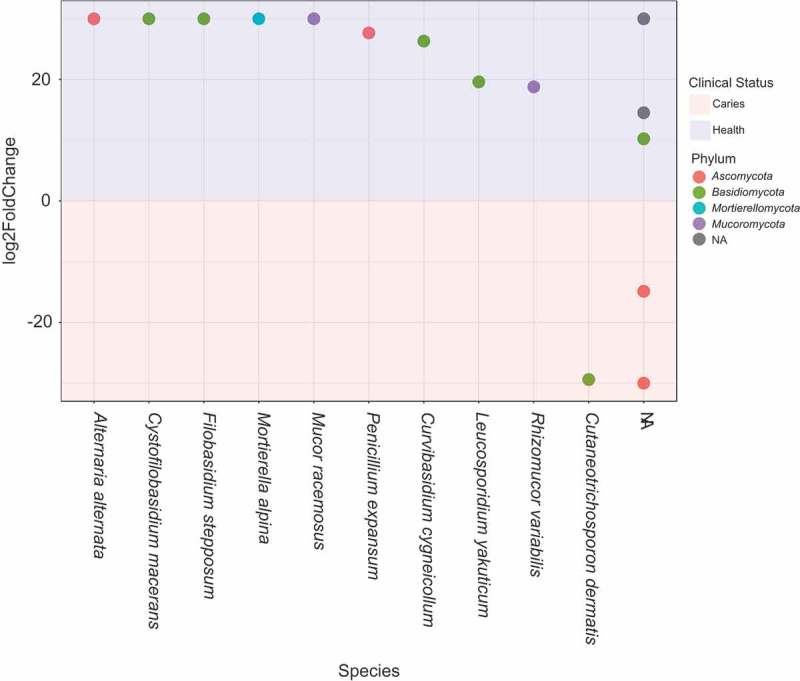
Differentially abundant species between caries and health. DESeq was used to investigate whether there were differentially abundant operational taxonomic units (OTUs) present in dental plaque samples in children with health and caries, when adjusting for age, sex and restorations (Phyloseq version 1.20.0). The figure shows species that were found to be significantly different (*p* < 0.001) between the two states. Positive log2-fold change values (above 0) indicate enriched species in children with health and negative log2-fold change values (below 0) indicate enriched species in children with dental caries. Abbreviation: Non-Assigned (NA).

Although overall diversity was similar, comparison of abundance of fungi in children with and without caries revealed a different profile of fungi. A differential abundance test, DESeq2 [48,49], was used to compare the abundance of OTUs in dental plaque samples from children with and without caries, when controlling for age, sex and the presence of restorations. We found 20 OTUs were differentially abundant between the two groups (see Figure 5). Of these 20, the majority of species (17) were significantly (*p* < 0.001) more abundant in children with healthy dentitions (see Supplementary Material 4). These included *Mucor racemosus, Filobasidium stepposum, Cystofilobasidium maceran, Penicillium expansum* and *Alternaria alternate proteae*.

The DESeq2 test can be negatively affected by differences in the number of sequences between samples [50]. As we had a large range of sequence depths between samples, we repeated the above analysis with restriction to samples with over 10,000 sequences. This produced consistent results with the unrestricted DESEq2 analysis (see Supplementary Figure 3 and Supplementary Material 5). The restricted compared to unrestricted analysis found the same species were more abundant in children with healthy dentitions, and revealed an even smaller number of fungal species were more abundant in caries. Only OTU 54 and 60 were significantly enriched in caries in both analyses.

## Discussion

The herein conducted analysis of the oral mycobiome is the first NGS study in children and highlights the potential impact of fungi on oral health state. The study found that children contain over 40 fungal species in their oral microbiome, which is dominated in terms of abundance and ubiquity by *C. albicans*. It also found that while *C. albicans* did not differ in abundance between children with healthy dentitions and caries, as it was expected, there was a significantly different profile of fungi between the health states. Healthy dentitions were associated with an enrichment of a wide range of fungi and caries was associated with a depletion of this diversity. While the specific species identified may be reflective of our investigated population, the pattern of reduced fungi in caries is more likely to be a translatable finding. Further research is now required to resolve how the change in fungal profile between a healthy and a caries state relates to changes in the bacterial community with caries development.

Current understanding of the oral mycobiome and how it impacts oral health in childhood is limited by a lack of culture-independent studies. At a broad-scale, we found a similar, but less diverse fungal profile when compared to investigations of the oral mycobiome in adult subjects [21–23]. For example, in comparison with the 46 fungal species/OTUs we identified in the childrens’ oral mycobiome, a study investigating 20 adults identified a total of 101 species across their samples [21]. Similar findings have been found when comparing bacterial diversity in the oral microbiome between child and adult populations [51]. The previously mentioned study of the adult oral mycobiome also used a different sample type, saliva (oral rinse), compared to the one herein that used dental plaque, which may have affected the diversity of fungi identified.

The current study also identified what made up the ‘core’ oral mycobiome in childhood, and found there was a considerable overlap in composition between this and the adult oral mycobiome. In both the children examined herein and previous studies of the adult oral mycobiome [21,22], *Candida, Malassezia, Saccharomyces* and *Cladosporium* species were identified as core members. The adult core oral mycobiome was also found to contain *Aureobasidium, Cystofilobasidium, Filobasidium* and *Penicillium* species [21,22]. These were identified in the childrens’ plaque samples, however were present in less than 50% of samples and not considered ‘core’ species. *R. mucilaginosa* was also found to be a core member in the childhood oral mycobiome. This species was amongst the most frequently isolated fungal species in a study examining the oral mycobiome in 40 adults [23].

Many of the fungi identified as a core of the childhood oral mycobiome are ubiquitous in the external environment and it is not unusual that they have been isolated from the oral cavity. For example, *Alternaria* and *Cladosporium* species have both been isolated from the airways and have been identified as a common airborne allergen associated with asthma [52,53]. *Saccharomyces cerevisiae* is commonly referred to as Baker’s yeasts and are frequently found as a harmless, transient fungus in the oral cavity and digestive tract [54]. *Rhodotorula* species are widespread environmental fungi but are known to be opportunistic pathogens frequently responsible for a number of infections, including infections during catheterisation, and in cases of endocarditis and peritonitis [55]. *Malassezia* have been identified as normal commensals of the skin, but are also known to be pathogens, responsible for an array of cutaneous diseases [56]. *M. globosa* has been identified in the sputum of patients with cystic fibrosis, and one of the main pathways for microorganisms to reach the airway is via the mouth [57].

The most dominant member of the core oral mycobiome in childhood identified herein was *C. albicans*, as has also been found in the adult oral mycobiome [21–23]. *Candida* species have the ability to colonise both hard (teeth) and soft (tongue, palate, buccal mucosa) tissues, and they are often found in both saliva and plaque biofilm [35]. High levels of *Candida* have been isolated from the mouths of children, especially when dental caries was present, and as such, it is not a surprise that *C. albicans* was identified so frequently in the current study [28]. Interestingly, *C. albicans* was ubiquitous in all samples regardless of whether there was caries present. The herein obtained findings did not show that *C. albicans* was enriched in children with dental caries, which differs from much research in this area to date.

While *C. albicans* was not found to increase in abundance with caries, it was found that a range of other fungal species differed between carious and healthy dentitions. The herein obtained results found 17 fungal species were significantly more abundant in children with healthy dentitions and very few were enriched in children with dental caries. Many of the species enriched in healthy dentitions included environmental fungi, previously identified in the airways, skin and oral cavity, such as *Alternaria alternata, M. globosa* and *M. racemosus* [52,53,56]. This is suggestive that children with healthy dentitions may have many commensal fungi present, however, when dental caries exists there may be an associated shift in the dental biofilm that makes it less favourable for the existence of a wide range of fungi. This may be due to there being lesser numbers of bacteria in children or, it could be due to the presence of bacteria suppressing fungi in children with caries. It is common for bacteria and fungi to co-inhabit many environments in and on the human body, and interactions between the two organisms can influence growth, behaviour and survival of both organisms [58,59]. Fungal viability may be reduced by certain bacterial factors such as nutrient depletion, secretion of anti-fungal molecules or transfer of toxins directly into the fungal cell [59].

Although our findings indicate that dental caries is associated with a reduction in abundance of a range of fungal species, the clinical significance of this is limited by the small sample size of 17 individuals studied herein and hence the findings should be considered preliminary. Drawing conclusions from these results is difficult, considering the small numbers involved that may mean the findings are specific to the investigated population. Furthermore, no replicas were included, reducing the robustness of our findings. In addition, a fragment of the ITS region was analysed as opposed to sequencing the whole ITS region. The combination of targeting a short DNA fragment with in-depth sequencing methods provided coverage of overall fungal community, and hence was useful for an exploratory, microbial-ecology study such as ours. However, this method is less able to resolve species level classifications compared with sequencing the whole ITS region. However, findings from our preliminary study indicate that further research is warranted to determine the role of fungi in oral health in childhood. In particular, NGS approaches provide the potential to investigate the interaction between bacteria and fungi in the oral microbiome and their combined contribution to dental caries and oral health. If fungi, in connection with bacteria in the oral microbiome, are found to play a significant role in the shift between a healthy dentition and dental caries, this may open up new treatment approaches, which target fungi-bacterial interactions for dental caries.

## Conclusions

A diverse range of fungal species was present in the oral cavity of healthy children, making an important contribution to the oral microbiome. *C. albicans* appeared to be frequently isolated from both caries-affected and caries-free dentitions, and was not more abundant in children with dental caries. Children without caries had a greater abundance of a wider diversity of fungi compared with those with caries. Due to the small size, future research is required to determine the clinical significance of these findings.

## Declarations

Written informed consent was obtained from the parents of all participants and ethical approval for the study was obtained from the University of Adelaide HREC (H-78-2003).

## Availability of data and material

The sequence data is available in the European Nucleotide Archive (ENA), accession number PRJEB28833.
