# Supplementary figures and images for: Preliminary study of the oral mycobiome of children with and without dental caries
Source: J Oral Microbiol. 2018 Oct 23;11(1):1536182. doi: 10.1080/20002297.2018.1536182 (PMC6225480; doi:10.1080/20002297.2018.1536182)

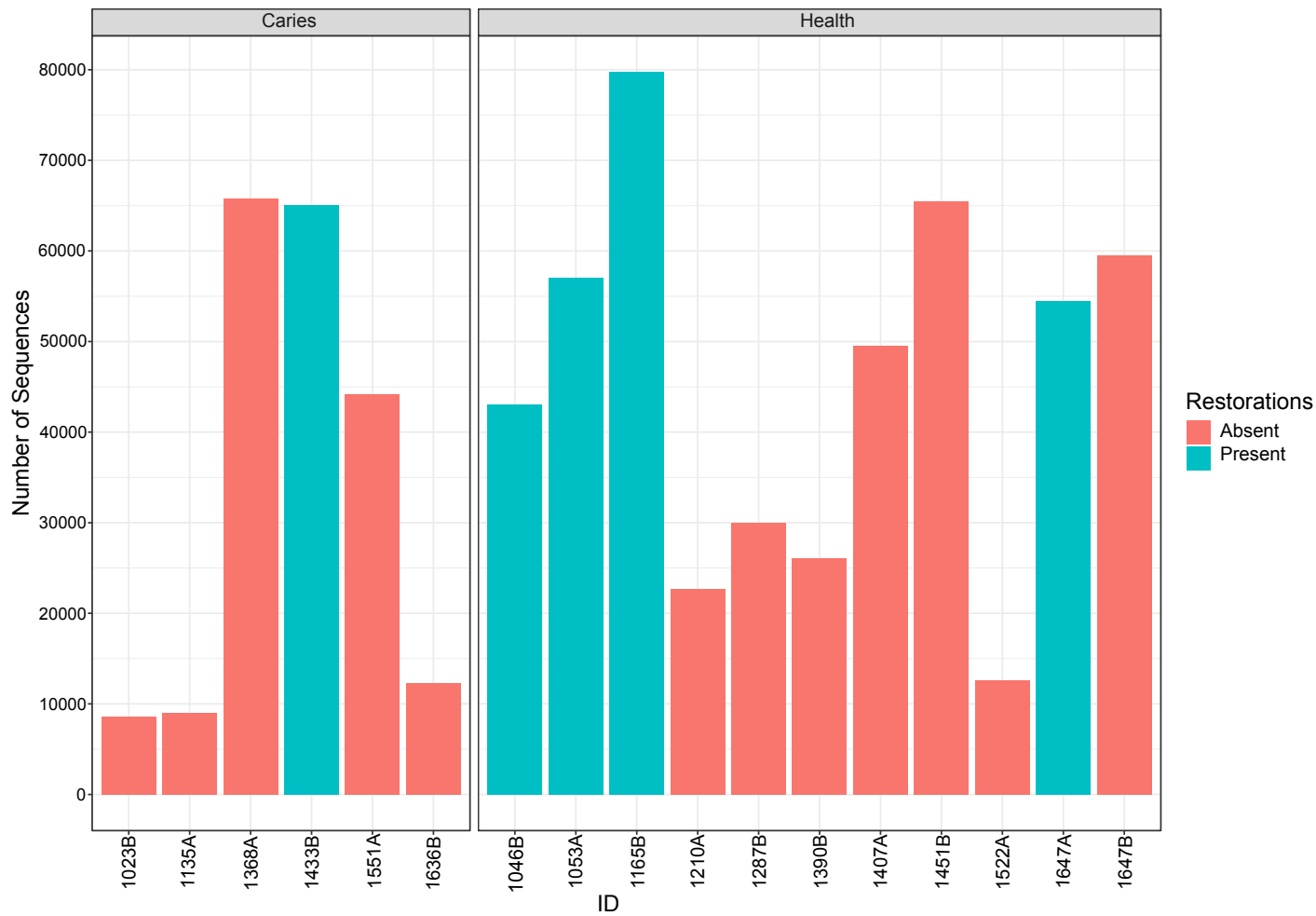

Supplement: Supplemental Material [file ZJOM_A_1536182_SM8541.zip › Supp_Mat/Supplementary Material Figure 1.pdf]

Abundance (%)

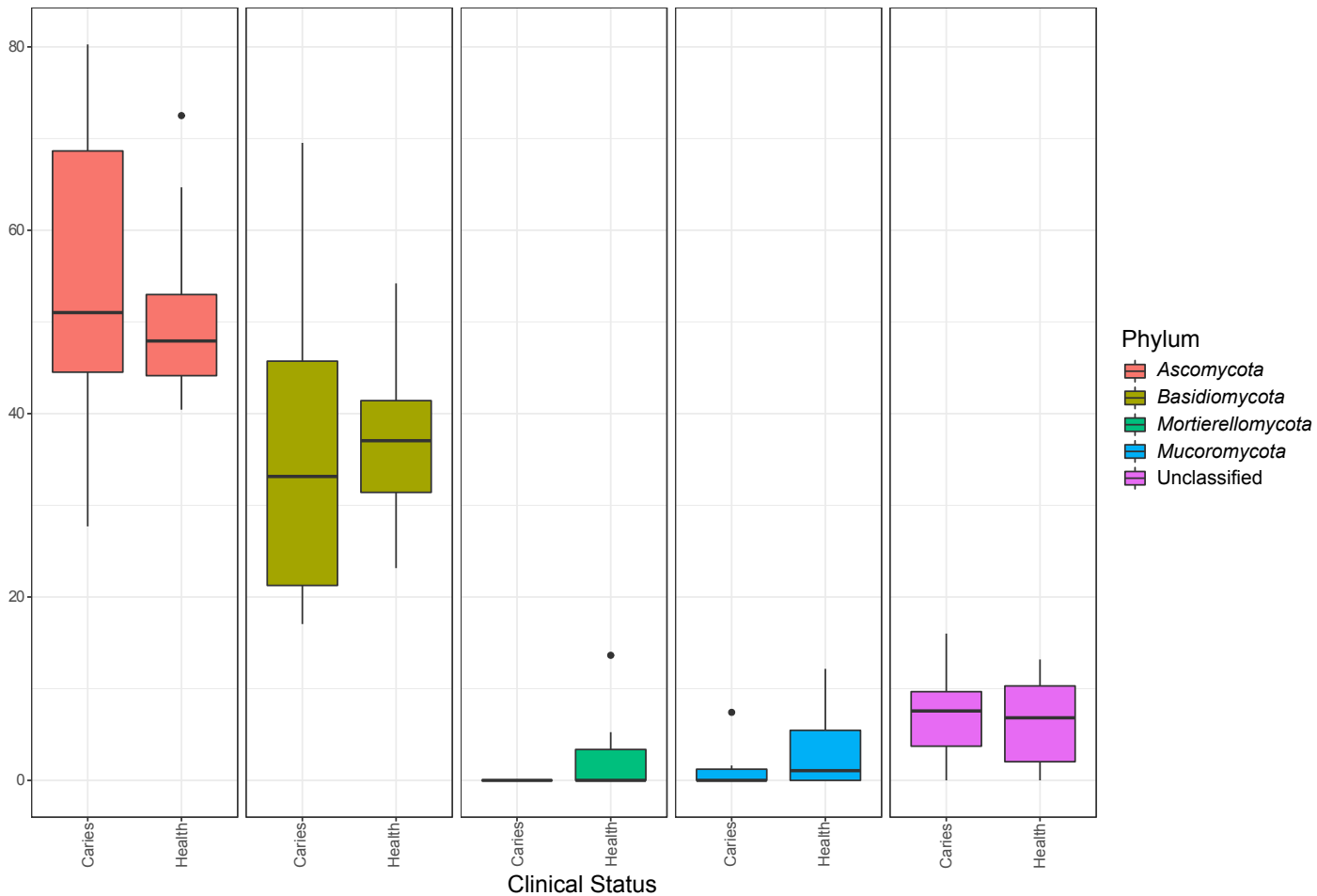

Supplement: Supplemental Material [file ZJOM_A_1536182_SM8541.zip › Supp_Mat/Supplementary Material Figure 2.pdf]

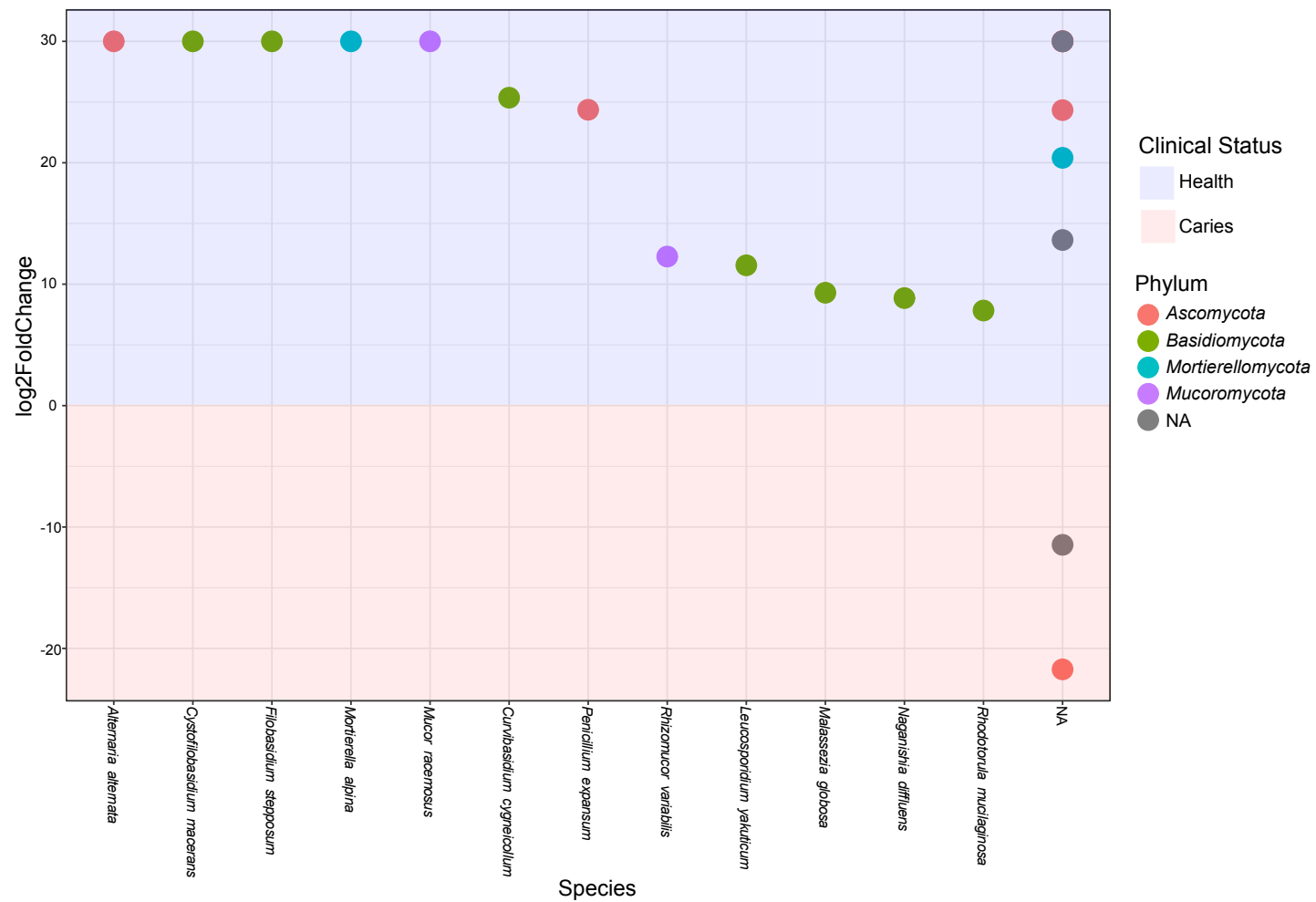

Supplement: Supplemental Material [file ZJOM_A_1536182_SM8541.zip › Supp_Mat/Supplementary Material Figure 3.pdf]
